# Supplementary material for: Effectiveness of Pre-Hospital Tourniquet in Emergency Patients with Major Trauma and Uncontrolled Haemorrhage: A Systematic Review and Meta-Analysis
Source: Int J Environ Res Public Health. 2021 Dec 6;18(23):12861. doi: 10.3390/ijerph182312861 (PMC8657739; doi:10.3390/ijerph182312861)
Supplement: Supplementary file 1 [file ijerph-18-12861-s001.zip › ijerph-1443262-supplementary/Supplementaries SR Tourniquet.pdf]

Supplement A. PICO Question and Search strategy

Review question: Is the use of pneumatic or mechanical tourniquets clinically and cost effective in improving outcomes in patients with haemorrhage in major trauma?

SEARCH STRATEGIES _CLINICAL QUESTION CQ4

1. **MEDLINE SEARCH TERMS**

Limitation of language: English, Spanish, Italian, French, German
No other filters

POPULATION

Medline search terms

1.	(trauma* or polytrauma*).ti,ab.
2.	((serious* or severe* or major or life threaten*) adj3 (accident* or injur* or fall*)).ti,ab.
3.	multiple trauma/
4.	wounds, gunshot/ or wounds, stab/ or accidents, traffic/ or accidental falls/ or blast injuries/ or accidents, aviation/
5.	((motor* or motorbike* or vehicle* or road or traffic or car or cars or cycling or bicycle* or automobile* or bike* or head on or pile up) adj3 (accident* or crash* or collision* or smash*)).ti,ab.
6.	(mvas or mva or rtas or rta).ti,ab.
7.	(stabbed or stabbing or stab or gunshot* or gun or gunfire or firearm* or bullet* or knife* or knives or dagger).ti,ab.
8.	or/1-7

INTERVENTION

Medline search terms

1.	tourniquets/
2.	tourniquet*.ti,ab.
3.	or/1-2

Excluded study designs and publication types

The following study designs and publication types were removed from retrieved results using **the NOT** operator.Added to search with operator NOT .The following search terms:

Medline search terms

1.	letter/
2.	editorial/
3.	news/
4.	exp historical article/
5.	anecdotes as topic/
6.	comment/
7.	case report/
8.	(letter or comment*).ti.
9.	or/1-8
10.	randomized controlled trial/ or random*.ti,ab.
11.	9 not 10

12.	animals/ not humans/
13.	exp animals, laboratory/
14.	exp animal experimentation/
15.	exp models, animal/
16.	exp rodentia/
17.	(rat or rats or mouse or mice).ti.
18.	or/11-17

2. **EMBASE SEARCH TERMS**

Limitation of language: english, spanish, Italian, German, French
No other filters.

POPULATION
major trauma population

Embase search terms

1.	(trauma* or polytrauma*).ti,ab.
2.	((serious* or severe* or major or life threaten*) adj3 (accident* or injur* or fall*)).ti,ab.
3.	multiple trauma/
4.	gunshot injury/ or stab wound/ or traffic accident/ or falling/ or blast injury/ or aircraft accident/
5.	((motor* or motorbike* or vehicle* or road or traffic or car or cars or cycling or bicycle* or automobile* or bike* or head on or pile up) adj3 (accident* or crash* or collision* or smash*)).ti,ab.

6.	(mvas or mva or rtas or rta).ti,ab.
7.	(stabbed or stabbing or stab or gunshot* or gun or gunfire or firearm* or bullet* or knife* or knives or dagger).ti,ab.
8.	or/1-7

INTERVENTION

Embase search terms

1.	exp tourniquet/
2.	tourniquet*.ti,ab.
3.	or/1-2

Excluded study designs and publication types

The following study designs and publication types were removed from retrieved results using **the NOT** operator.Added to search with operator NOT , the following search terms:

Embase search terms

1.	letter.pt. or letter/
2.	note.pt.
3.	editorial.pt.
4.	case report/ or case study/
5.	(letter or comment*).ti.
6.	or/1-5
7.	randomized controlled trial/ or random*.ti,ab.

8.	6 not 7
9.	animal/ not human/
10.	nonhuman/
11.	exp animal experiment/
12.	exp experimental animal/
13.	animal model/
14.	exp rodent/
15.	(rat or rats or mouse or mice).ti.
16.	or/8-15

3. COCHRANE SEARCH TERMS

Limitation of language: English, Spanish, Italian, French, German
No other filters

POPULATION

Cochrane search terms

#1.	MeSH descriptor: [multiple trauma] this term only
#2.	(trauma* or polytrauma*):ti,ab
#3.	((serious* or severe* or major) near/3 (accident* or injur* or fall*)):ti,ab
#4.	MeSH descriptor: [wounds, gunshot] this term only
#5.	MeSH descriptor: [wounds, stab] this term only
#6.	MeSH descriptor: [accidents, traffic] this term only
#7.	MeSH descriptor: [accidental falls] this term only
#8.	MeSH descriptor: [blast injuries] this term only

#9.	MeSH descriptor: [accidents, aviation] this term only
#10.	((motor* or motorbike* or vehicle* or road or traffic or car or cars or cycling or bicycle* or automobile* or bike*) near/3 (accident* or crash* or collision* or smash*)):ti,ab
#11.	(mvas or mva or rtas or rta):ti,ab
#12.	(stabbed or stabbing or stab or gunshot or gun or gunfire or firearm* or bullet or knife* or knives or dagger or shot):ti,ab
#13.	{or #1-#12}

INTERVENTION

Cochrane search terms

#1.	MeSH descriptor: [tourniquets] this term only
#2.	tourniquet*:ti,ab
#3.	{or #1-#2}

Supplement B. List of excluded studies with reasons

Studies excluded from the clinical review (UPDATE 2015-2020)

N°	REFERENCES	REASON OF EXCLUSION
1	Zietlow JM, Zietlow SP, Morris DS, Berns KS, Jenkins DH. Prehospital Use of Hemostatic Bandages and Tourniquets: Translation From Military Experience to Implementation in Civilian Trauma Care. J SpecOperMed. 2015;15(2):48–53	OUT OF SCOPE
2	Shackelford SA, Del Junco DJ, Powell-Dunford N, et al. Association of Prehospital Blood Product Transfusion During Medical Evacuation of Combat Casualties in Afghanistan With Acute and 30-Day Survival. JAMA. 2017;318(16):1581–1591. doi:10.1001/jama.2017.15097	POPULATION
3	Schauer SG, Naylor JF, April MD, et al. The Prehospital Trauma Registry Experience With Intraosseous Access. J SpecOperMed. 2019;19(1):52–55.	NOT AVAILABLE
4	Schauer SG, April MD, Naylor JF, et al. Prehospital Application of Hemostatic Agents in Iraq and Afghanistan. PrehospEmerg Care. 2018;22(5):614–623. doi:10.1080/10903127.2017.1423140	POPULATION
5	Mawhinney AC, Kirk SJ. A systematic review of the use of tourniquets and topical haemostatic agents in conflicts in Afghanistan and Iraq. J R NavMedServ. 2015;101(2):147–154.	NOT AVAILABLE
6	Duignan KM, Lamb LC, DiFiori MM, Quinlavin J, Feeney JM. Tourniquet use in the prehospital setting: Are they being used appropriately?.Am J DisasterMed. 2018;13(1):37–43. doi:10.5055/ajdm.2018.0286	NOT AVAILABLE
7	Beaucreux C, Vivien B, Miles E, Ausset S, Pasquier P. Application of tourniquet in civilian trauma: Systematic review of the literature. AnaesthCrit Care PainMed. 2018;37(6):597–606.	WRONG COMPARISON
9	Kauvar DS, Dubick MA, Walters TJ, Kragh JF Jr. Systematic review of prehospital tourniquet use in civilian limb trauma. J Trauma Acute Care Surg. 2018;84(5):819–825.	WRONG COMPARISON

8	Scerbo MH, Mumm JP, Gates K, et al. Safety and Appropriateness of Tourniquets in 105 Civilians. PrehospEmerg Care. 2016;20(6):712–722.	OUT OF SCOPE
10	vanOostendorp SE, Tan EC, Geeraedts LM Jr. Prehospital control of life-threatening truncal and junctional haemorrhage is the ultimate challenge in optimizing trauma care; a review of treatment options and their applicability in the civilian trauma setting. Scand J Trauma ResuscEmergMed. 2016;24(1):110	POPULATION
12	Hossfeld B, Lechner R, Josse F, et al. PrähospitaleAnwendung von Tourniquets beilebensbedrohlichenExtremitätenblutungen :EinesystematischeÜbersichtsarbeit [Prehospital application of tourniquets for life-threatening extremity hemorrhage : Systematic review of literature]. Unfallchirurg. 2018;121(7):516–529.	OUT OF SCOPE
13	Meizoso JP, Valle EJ, Allen CJ, et al. Decreased mortality after prehospital interventions in severely injured trauma patients. J Trauma Acute Care Surg. 2015;79(2):227–231.	POPULATION
14	Schauer SG, April MD, Hill GJ, Naylor JF, Borgman MA, De Lorenzo RA. Prehospital Interventions Performed on Pediatric Trauma Patients in Iraq and Afghanistan. PrehospEmerg Care. 2018;22(5):624–629.	OUT OF SCOPE
15	El Sayed MJ, Tamim H, Mailhac A, Mann NC. Trends and Predictors of Limb Tourniquet Use by Civilian Emergency Medical Services in the United States. PrehospEmerg Care. 2017;21(1):54–62.	INTERVENTION
16	Schroll R, Smith A, McSwain NE Jr, et al. A multi-institutional analysis of prehospital tourniquet use. J Trauma Acute Care Surg. 2015;79(1):10–14.	POPULATION
17	Inaba K, Siboni S, Resnick S, et al. Tourniquet use for civilian extremity trauma. J Trauma Acute Care Surg. 2015;79(2):232–333.	STUDY DESIGN
18	Leonard J, Zietlow J, Morris D, et al. A multi-institutional study of hemostatic gauze and tourniquets in rural civilian trauma. J Trauma Acute Care Surg. 2016;81(3):441–444.	STUDY DESIGN
19	Schauer SG, April MD, Fisher AD, Cunningham CW, Gurney J. Junctional Tourniquet Use During Combat Operations in Afghanistan: The Prehospital Trauma Registry Experience. J SpecOperMed. 2018;18(2):71–74.	OUT OF SCOPE

20	Cantle PM, Hurley MJ, Swartz MD, Holcomb JB. Methods for Early Control of Abdominal Hemorrhage: An Assessment of Potential Benefit. J SpecOperMed. 2018;18(2):98–104.	OUT OF SCOPE

Supplement C. Internal validity

All studies show poor reporting of outcome data by not indicating the follow-up of the assessment.

	Selection				Comparability	Outcome			Judgement	
**Cohort study**	Representativeness of the exposed cohort	Selection of the non exposed cohort	Ascertainment of exposure	Demonstration that outcome of interest was not present at start of study	Comparability of cohorts on the basis of the design or analysis	Assessment of outcome	Was follow-up long enough for outcomes to occur	Adequacy of follow up of cohorts	Total	Quality
**McNickle et al. (2019)**		*	*		*	*			4	Fair
**Scerbo et al. (2017)**	*	*	*		*	*	*		6	Good
**Smith et al. (2018)**	*	*	*		*	*	*		6	Good
**Teixeira et al. (2018)**		*	*		*	*	*		5	Good

§ Outcomes may have been influenced by time.

Thresholds for converting the Newcastle-Ottawa scales to AHRQ standards (good, fair, and poor):

Good quality: 3 or 4 stars in selection domain AND 1 or 2 stars in comparability domain AND 2 or 3 stars in outcome/exposure domain

Fair quality: 2 stars in selection domain AND 1 or 2 stars in comparability domain AND 2 or 3 stars in outcome/exposure domain

Poor quality: 0 (zero) or 1 star in selection domain OR 0 stars in comparability domain OR 0 or 1 stars in outcome/exposure domain

Cohort studies

Note: A study can be awarded a maximum of one star for each numbered item within the Selection and Outcome categories. A maximum of two stars can be given for Comparability

Selection

1) Representativeness of the exposed cohort
   - a) truly representative of the average population in the community *
   - b) somewhat representative of the average population in the community *
   - c) selected group of users eg nurses, volunteers
   - d) no description of the derivation of the cohort

2) Selection of the non exposed cohort
   - a) drawn from the same community as the exposed cohort *

    b) drawn from a different source
    c) no description of the derivation of the non exposed cohort
3) Ascertainment of exposure
    a) secure record (eg surgical records) *
    b) structured interview [illegible]
    c) written self report
    d) no description
4) Demonstration that outcome of interest was not present at start of study
    a) yes *
    b) no

Comparability

1) Comparability of cohorts on the basis of the design or analysis
    a) study controls for the most important factor*
    b) study controls for any additional factor *
    c) no control for confounding performed

Outcome

1) Assessment of outcome
    a) independent blind assessment *
    b) record linkage *
    c) self report
    d) no description
2) Was follow-up long enough for outcomes to occur
    a) yes (select an adequate follow up period for outcome of interest) *
    b) no
3) Adequacy of follow up of cohorts
    a) complete follow up - all subjects accounted for *
    b) subjects lost to follow up unlikely to introduce bias - small number lost - >70 % follow up, or description provided of those lost) *
    c) follow up rate <70% and no description of those lost
    d) no statement

Supplement D. Summary of Findings using GRADE approach - Tables

Certainty assessment							№ of patients		Effect		Certainty	Importance
№ of studies	Study design	Risk of bias	Inconsistency	Indirectness	Imprecision	Otherconsiderations	Tourniquets	no tourniquets (adjusted data)	Relative (95% CI)	Absolute (95% CI)		
**Overall cause mortality (follow-up not reported)**												
3	Observationalstudies	serious[a,b]	notserious	serious[c]	serious[d]	suspected [h]	22/448 (4.9%)	16/175 (9.1%)	OR 0.47 (0.19 to 1.16)	46 fewer per 1.000 (from 73 fewer to 13 more)	⨁◯◯◯ VERY LOW	CRITICAL
**Mortality (cause of death-haemorrhage)**												
1	Observationalstudies	serious[b,e]	notserious	serious[c]	notserious	none	8/252 (3.2%)	4/29 (13.8%)	OR 0.22 (0.06 to 0.80)	104 fewer per 1.000 (from 128 fewer to 24 fewer)	⨁◯◯◯ VERY LOW	CRITICAL
**Health related quality of life**												
No studies	-	-	-	-	-	-	-	-	-	-	-	CRITICAL
**ICU length of stay (assessed with: Days)**												
1	Observationalstudies	serious[a,b,e]	notserious	serious[c]	notserious	suspected [h]	-	-	-	-	-	CRITICAL
**ICU free days (assessed with: Days)**												
1	Observationalstudies	serious[a,b,e]	notserious	serious[c]	notserious	none	69	69	-	MD 0.9 lower (2.71 lower to 0.91 higher)	⨁◯◯◯ VERY LOW	CRITICAL
**Blood product use - 1) PRBC**												

Certainty assessment							№ of patients		Effect		Certainty	Importance
№ of studies	Study design	Risk of bias	Inconsistency	Indirectness	Imprecision	Otherconsiderations	Tourniquets	no tourniquets (adjusted data)	Relative (95% CI)	Absolute (95% CI)		
2	Observationalstudies	serious[a,b,e]	veryserious[f]	serious[c]	serious[g]	suspected [h]	196	146	-	MD 3.28 lower (11.22 lower to 4.66 higher)	⊕◯◯◯ VERY LOW	CRITICAL
**Blood product use - 2) Platelets**												
No studies	-	-	-	-	-	-	-	-	-	-	-	CRITICAL
**Blood product use - 3) Plasma**												
1	Observationalstudies	serious[a,b,e]	notserious	serious[c]	notserious	suspected [h]	127	77	-	MD 4.8 lower (5.61 lower to 3.99 lower)	⊕◯◯◯ VERY LOW	CRITICAL
**Blood product use -4) Cryoprecipitate**												
No studies	-	-	-	-	-	-	-	-	-	-	-	CRITICAL
**Adverseevents - 1.1) initialamputation**												
1	Observationalstudies	serious[b,e]	notserious	serious[c]	notserious	none	16/69 (23.2%)	4/69 (5.8%)	OR 4.91 (1.55 to 15.56)	174 more per 1.000 (from 29 more to 431 more)	⊕◯◯◯ VERY LOW	CRITICAL
**Adverseevents – 1.2) delayedamputation**												
2	Observationalstudies	serious[a,b,e]	notserious	serious[c]	serious[d,g]	suspected [h]	7/180 (3.9%)	10/130 (7.7%)	OR 0.45 (0.02 to 11.28)	41 fewer per 1.000 (from 75 fewer to 408 more)	⊕◯◯◯ VERY LOW	CRITICAL
**Adverseevents - 2) nervepalsy**												
1	Observationalstudies	serious[b,e]	notserious	serious[c]	notserious	none	8/127 (6.3%)	2/77 (2.6%)	OR 2.52 (0.52 to 12.20)	37 more per 1.000 (from 12 fewer to 219 more)	⊕◯◯◯ VERY LOW	CRITICAL

Certainty assessment							№ of patients		Effect		Certainty	Importance
№ of studies	Study design	Risk of bias	Inconsistency	Indirectness	Imprecision	Otherconsiderations	Tourniquets	no tourniquets (adjusted data)	Relative (95% CI)	Absolute (95% CI)		
**Adverseevents - 3) renalfailure**												
1	Observationalstudies	serious[a,b,e]	notserious	serious[c]	notserious	none	4/69 (5.8%)	6/69 (8.7%)	OR 0.65 (0.17 to 2.44)	29 fewer per 1.000 (from 71 fewer to 102 more)	⨁◯◯◯ VERY LOW	CRITICAL

Certaintyassessment							№ of patients		Effect		Certainty	Importance
№ of studies	Study design	Risk of bias	Inconsistency	Indirectness	Imprecision	Otherconsiderations	Tourniquets	no tourniquets (adjusted data)	Relative (95% CI)	Absolute (95% CI)		
adverse events - 4) increased bleeding: procedure to control bleeding												
1	Observationalstudies	serious[a,c]	notserious	serious[b]	notserious	none	27/127 (21.3%)	32/77 (41.6%)	OR 0.38 (0.20 to 0.72)	203 fewer per 1.000 (from 291 fewer to 77 fewer)	⨁◯◯◯ VERY LOW	CRITICAL
Time to definitive control of haemorrhage												
No studies									notestimable		-	IMPORTANT
Patient-reported outcomes (psychological wellbeing)												
No studies									notestimable		-	IMPORTANT

*the absolute effect comes from crude estimate whereas the relative effect comes from adjusted estimate

CI: Confidence interval; OR: Odds ratio; MD: Mean difference

Explanations

a. Representativeness of the exposed cohort

b. Adequacy of follow up of cohorts

c. USA setting

d. number of events <200

e. Demonstration that outcome of interest was not present at start of study

f. I2>75%

g. Confidence intervals crossed the line of no difference with plausible effects in favor to the experimental group

h. we did not considered one study for unclear reporting outcome data
